# Enhancing In Vivo Electroporation Efficiency through Hyaluronidase: Insights into Plasmid Distribution and Optimization Strategies

**DOI:** 10.3390/pharmaceutics16040547

**Published:** 2024-04-17

**Authors:** Debnath Maji, Verónica Miguela, Andrew D. Cameron, Delcora A. Campbell, Linda Sasset, Xin Yao, Andy T. Thompson, Carleigh Sussman, David Yang, Robert Miller, Marek M. Drozdz, Rachel A. Liberatore

**Affiliations:** 1 RenBio Inc., Long Island City, New York, NY 11101, USA; 2 Instituto de Neurociencias, Consejo Superior de Investigaciones Científicas—Universidad Miguel Hernández de Elche, Sant Joan d’Alacant, 03550 Alicante, Spain; 3 Department of Genetics, Albert Einstein College of Medicine, Bronx, NY 10461, USA

**Keywords:** hyaluronidase, plasmid DNA, electroporation, bioimpedance, extracellular matrix, transfection, gene electrotransfer

## Abstract

Electroporation (EP) stands out as a promising non-viral plasmid delivery strategy, although achieving optimal transfection efficiency in vivo remains a challenge. A noteworthy advancement in the field of in vivo EP is the application of hyaluronidase, an enzyme with the capacity to degrade hyaluronic acid in the extracellular matrix, which thereby enhances DNA transfer efficiency by 2- to 3-fold. This paper focuses on elucidating the mechanism of hyaluronidase’s impact on transfection efficiency. We demonstrate that hyaluronidase promotes a more uniform distribution of plasmid DNA (pDNA) within skeletal muscle. Additionally, our study investigates the effect of the timing of hyaluronidase pretreatment on EP efficiency by including time intervals of 0, 5, and 30 min between hyaluronidase treatment and the application of pulses. Serum levels of the pDNA-encoded transgene reveal a minimal influence of the hyaluronidase pretreatment time on the final serum protein levels following delivery in both mice and rabbit models. Leveraging bioimpedance measurements, we capture morphological changes in muscle induced by hyaluronidase treatment, which result in a varied pDNA distribution. Subsequently, these findings are employed to optimize EP electrical parameters following hyaluronidase treatment in animal models. This paper offers novel insights into the potential of hyaluronidase in enhancing the effectiveness of in vivo EP, as well as guides optimized electroporation strategies following hyaluronidase use.

## 1. Introduction

Plasmid DNA (pDNA) serves as a versatile vehicle for introducing foreign genetic material into cells, providing researchers with the means to study gene function, develop gene therapies, and express proteins for various applications. The success of these endeavors crucially hinges on the effectiveness of the chosen method for delivering pDNA into the target cells. The transfer of genetic material into cells has been facilitated by an array of methods, each with its unique strengths and limitations. Viral vectors, chemical transfection agents, and physical methods such as microinjection and electroporation have emerged as prominent techniques [1,2,3].

Chemical methods, including lipofection, calcium phosphate transfection, and polymeric carrier-mediated transfection, utilize synthetic or natural compounds to aid in the cellular uptake of pDNA. While these methods are versatile and relatively simple to employ in vitro, issues such as cytotoxicity, variable transfection efficiency, and the need for optimization in specific cell types underscore their limitations [4,5]. In addition to these challenges associated with in vitro transfection methods, in vivo transfection presents a new set of complexities for researchers. The in vivo environment introduces physiological barriers, including immune responses, enzymatic degradation, and complex biological interactions, which can significantly impact the effectiveness of synthetic or natural compounds used for transfection. Viral vectors, such as adeno-associated virus (AAV) and lentiviruses, leverage the natural infection machinery of viruses and have gained prominence recently for their efficiency in gene delivery. However, concerns over immunogenicity, limited cargo capacity, and potential integration into the host genome pose challenges [6,7].

Among the diverse plasmid delivery methods available, electroporation stands out as a versatile and potent approach for facilitating the transfer of pDNA into target cells. Electroporation has become more relevant due to its ability to induce transient permeabilization of cell membranes via the application of electric fields, allowing for the efficient uptake of pDNA into those cells both in vitro and in vivo [8,9]. However, despite its efficiency, electroporation is not without limitations. Cellular stress induced by electric pulses may lead to reduced cell viability and altered cellular functions [10,11]. The potential for cell damage and the requirement for high-voltage pulses in certain applications underscore the need for a nuanced understanding of these drawbacks to optimize and refine the technique [12].

Optimizing electroporation is paramount to realizing its full potential in pDNA delivery, especially in vivo. It is imperative to strike a balance between maximizing DNA uptake and ensuring cell viability. Parameters such as pulse duration, voltage, and the number of pulses play crucial roles in determining the efficiency and viability of the process [13]. Additionally, the electroporation buffer composition and the type of electrode used are critical factors that can influence the outcome [14]. Moreover, advancements in electrode design, pulse waveform optimization, and the development of specialized electroporation instruments contribute to the ability to refine and customize the electroporation process for specific cell types and applications [15]. Greater understanding of the underlying mechanisms of electroporation and its interaction with cellular membranes has led to the adoption of innovative approaches aimed at enhancing its efficiency.

The use of hyaluronidase, an enzyme, stands out as a significant advancement in the field of electroporation in vivo. Its unique ability to break down hyaluronic acid (HA), a major component of the extracellular matrix (ECM), paves the way for improved DNA transfer efficiency. By temporarily reducing the barriers presented by the ECM, hyaluronidase enhances the accessibility of cells to the injected pDNA, thereby amplifying the efficiency of electroporation-induced transfection [16]. 

Herein, we aim to shed new light on the optimized use of hyaluronidase as a factor in improving pDNA uptake on a cellular level in vivo. We also investigate its impact on electroporation outcomes in animal models and offer insights into the optimization of parameters that may catalyze further improvements in the field of pDNA transfer using electroporation.

## 2. Materials and Methods

### 2.1. pDNA Constructs

DNA encoding the IgG heavy chain (HC) and light chain (LC) of S139/1 IgG [17], 1219 (GenBank: QIQ28227 and QIQ28233; unpublished), or EBV321 IgG [18] were cloned into the pCBAINA, a gWiz-derived expression construct. The backbone of pCBAINA includes a cytomegalovirus (CMV) enhancer, chicken beta actin promoter, CMV intron A, bovine growth hormone polyadenylation (BGH-PolyA) sequence, and kanamycin resistance gene. Antibody (Ab) sequences were codon optimized by GeneArt (Thermo Fisher Scientific, Waltham, MA, USA) and synthesized by Twist Bioscience (San Francisco, CA, USA). 

### 2.2. Animals

All animal procedures were performed at Mispro facilities and overseen by the Mispro Institutional Animal Care and Use Committee (IACUC), with the animals cared for in accordance with all federal and state regulations. The mice were housed in disposable, individually ventilated, filter-top cages (Innocage®, Innovive Inc., San Diego, CA, USA), and the caging space, temperature, and humidity for the rabbits were maintained in accordance with the Animal Welfare Act and the Guide for the Care and Use of Laboratory Animals recommendations. 

Mouse experiments were conducted on 6–9-week-old BALB/c female mice (BALB/cAnNCrl) with an approximate weight of 17–20 g. Rabbit experiments were conducted on 8–12-week-old New Zealand White female rabbits with an approximate starting weight of 1.8–2.4 kg. All animals were procured from Charles River Laboratories (Charles River, Wilmington, MA, USA).

### 2.3. Intramuscular Electroporation

Electroporation was performed in the tibialis anterior (TA) muscle in mice. Recombinant Hylenex® (hyaluronidase human injection, Halozyme Therapeutics, San Diego, CA, USA) was chosen as the enzyme to study the effects of hyaluronidase. Hylenex® is supplied as an injectable reconstituted in saline at 150 U/mL. In experiments involving pretreatment with hyaluronidase, the target muscle was injected with 20 µL of 150 U/mL Hylenex, 5–30 min prior to pDNA electrotransfer. The dose and pretreatment time ranges were guided by a combination of previously conducted internal studies and pertinent literature [19,20]. For the pDNA electrotransfer, a 20 μL mixture of pCBAINA-HC and pCBAINA-LC pDNA of equal mass was formulated in 0.5 × 0.9% saline at a final concentration of 0.25 μg/μL and injected using a 29-gauge syringe (324702, Becton Dickinson, Franklin Lakes, NJ, USA). The depth of the injection was fixed at 1.75 mm using a 3D printed stopper. The pDNA injection was immediately followed by electroporation using an in-house-developed needle-based electroporator. The electroporator consists of four 0.3 mm diameter gold acupuncture needle electrodes (AMG-3030, Asiamed, Pullach, Germany), each at the corner of a 2 × 2 mm square. A series of pulses at 150 V/cm was applied using a RenBio custom-built generator (RenBio Inc., Queens, NY, USA). Current and voltage readings were obtained via a differential voltage probe and current probe, respectively, and recorded via a Red Pitaya. 

For rabbits, electroporation was performed in the vastus lateralis muscle. For the pDNA electrotransfer, an 800 μL mixture of HC- and LC-encoding pDNA of equal mass and with a final concentration of 0.5 μg/μL was constituted in 0.5 × 0.9% saline. For pDNA electrotransfer involving coformulation with Hylenex, 400 μL of a mixture of HC- and LC-encoding pDNA of equal mass in DI water was mixed with 400 μL of Hylenex (150 U/mL) to a final concentration of 0.5 µg/µL. The rabbit electroporator device consisted of six partially insulated 23-gauge stainless steel electrodes with the pDNA injection in the center of the electrode array. A series of pulses at 150 V/cm was applied using a RenBio custom-built generator. Current and voltage readings were obtained via a differential voltage probe and current probe, respectively, and recorded via a Red Pitaya. 

### 2.4. Bioimpedance Measurements and Fitting

Tissue impedance for both mice and rabbits was recorded using the electrodes of the electroporation device. An impedance analyzer (Agilent 4294A, Agilent Inc., Santa Clara, CA, USA) was used with a custom-made printed circuit board (PCB) test fixture and 1 m long, four-terminal extension cable. Measurement sweeps of the Z and θ parameters were conducted in a frequency range of 100 Hz to 1 MHz. The setup was calibrated using custom-made PCBs with fixed impedance. Impedance sweeps were performed before injection of the plasmid, after injection of plasmid, and after application of electrical pulses. 

The utilization of electrical equivalent circuits aims to establish a link between impedance measurements and a behavioral or physiologically derived electrical circuit model of the tissue. This approach involves the selection of a suitable circuit model and determining the circuit parameters that most accurately correspond to the experimental data. A commonly employed circuit in the field of biology and biomedicine is the Cole–Cole model [21], which consists of three components including two resistors and a constant phase element, as represented in Equation (1) [22]. Equation (2) defines the electrode polarization effect that happens at low frequencies and dominates the impedance readings below a few KHz. The electrode polarization effect is an artifact and can be affected by external factors not related to the tissue. The electrode polarization is modeled as a constant phase element model and the impedance is in series with the tissue impedance. The final Equation (3) depicts the total measured impedance, recorded as a series sum of the tissue impedance and the impedance due to electrode polarization. 

To model and interpret the experimentally acquired data to the equivalent circuit, a fitting process was utilized. The fitting process employed a non-linear least-squares procedure, iteratively applied to both the real and imaginary parts of the obtained impedance data. This approach ensured a robust representation of the bioimpedance data, facilitating a comprehensive analysis and interpretation in alignment with the Cole–Cole model. Post-fitting, parameters obtained such as *R_e_*, *R_i_*, *α*, and *C* were compared for different experimental conditions.

(1) $$Y_{tissue}=\frac{1}{R_{e}}+\frac{1}{R_{i}+\frac{1}{{j\omega }^{\alpha }C}}$$

(2) $$Ycpe=A\cdot (j{\omega )}^{acpe}$$

(3) $$Z=\frac{1}{Y_{tissue}}+\frac{1}{Ycpe}$$

### 2.5. PNA-pDNA Labeling

A peptide nucleic acid (PNA) conjugated to AlexaFluor 647 (A647) was used as a fluorescent pDNA marker, designed as previously reported [23]. The PNA sequence used was KK-TCTCTCTC-O-O-O-JTJTTJTJT-KK, where J is a pseudoisocytosine and O is an 8-amino 3,6-dioxaoctanoic acid linker. 

pDNA was labeled by annealing with the A647-labeled PNA as described previously [24,25]. Briefly, 1.2 mg of pDNA was mixed with 57.6 μL of 50 μM A647-PNA (PNA Bio, Thousand Oaks, CA, USA) in 1x TAE (BM-250, Boston Bioproducts, Milford, MA, USA), in a final volume of 800 μL. This mix was then split evenly into two light-resistant containers and incubated for 4 h at 40 °C. Post-incubation, 40 μL of 3 M NaAcetate, pH 5.2 (S7899-500ML, Sigma-Aldrich, St. Louis, MO, USA) and 1 mL of cold 70% EtOH (861263, Carolina Biological Supply, Burlington, NC, USA) were added to each mix, incubated on ice for 10 min, and centrifuged at 21,000× *g* for 5 min at 4 °C. After the spin cycle, the pellet was washed with 500 µL of 70% EtOH, followed by another 5 min spin at 21,000× *g* and 4 °C. The supernatant was discarded, and the remaining pellet was left to dry for approximately 10 min. Finally, 400 μL of DI water and 400 μL of saline (Z1377, Cytiva, Marlborough, MA, USA) were added to the pellet and left to dissolve overnight at 4 °C. The final PNA-labeled pDNA yield was verified using a NanoDrop® spectrophotometer. 

### 2.6. Blood Collection, Tissue Processing, and Sectioning

Blood sample collection was performed at multiple timepoints post-electrotransfer, as indicated in the figures. Blood was processed to serum and stored at −20 °C for further analysis.

For the PNA studies in mice, TA sites were injected with 20 μL of PNA-labeled pDNA at 0.333 mg/mL, immediately after which the animals were euthanized and the target tissue was excised. Extracted tissues were fixed in 10% paraformaldehyde (HT501128-4L, Sigma-Aldrich, St. Louis, MO, USA) for approximately 72 h, after which they were rinsed with 1x PBS (BM-220, Boston Bioproducts, Milford, MA, USA) and securely stored in 1x PBS in light-resistant containers.

For processing, each sample was placed in a potting mold. A 4% *w*/*v* Agarose II (A9918-100G, Sigma-Aldrich, St. Louis, MO, USA) gel was evenly poured over each sample and allowed to set at room temperature for an hour. Agarose blocks were sliced into 0.5–1.0 mm sections and then stored in 1x PBS. Sections were further processed by HistoWiz (Queens, NY, USA) for embedding, sectioning, staining, and imaging. Briefly, samples were paraffin wax-embedded, sectioned to 4 μm, mounted on slides, counterstained with DAPI, and subjected to imaging. 

### 2.7. Microscopy and Image Acquisition

Immunofluorescence (IF) was performed at HistoWiz Inc. following a standard operating procedure under GLP regulations and a fully automated workflow. Mouse tissue was sectioned (4 μm) transversely to the orientation of muscle fibers and mounted on positively charged slides. DAPI counterstaining to visualize nuclei was performed on the Bond Rx Auto Stainer (Leica Biosystems, Wetzlar, Germany) using Spectral Dapi (Akoya FP1490, Akoya Biosciences, Marlborough, MA, USA). Following staining, slides were cover slipped with Prolong Diamond Antifade Mounting Medium (Thermo P36961) to prevent bleaching and signal loss. Finally, slides were imaged with a DAPI filter and a Cy5-MSI filter aligned to capture A647. Gain and exposure were kept consistent across all slides with DAPI at 0.72 ms and 6.2 ms for Cy5-MSI. 

### 2.8. ELISA Analysis

The levels of antibodies in the serum samples were quantified by ELISA. Corning 9018 plates were coated with the appropriate target antigen overnight at 4 °C in 0.2 M BupH carbonate–bicarbonate buffer, pH 9.4 (Thermo Scientific). For the EBV321 ELISA, human/cynomolgus VEGF/VEGFA/VEGF165-20 protein (Sino Biological #11066-HNAH, Beijing, China) antigen at a concentration of 8 µg/mL was used; for the 1219 ELISA, SARS-CoV-2 (2019-nCoV) spike RBD-His recombinant protein (Sino Biological #40592-V08H) was used as the antigen at a concentration of 2 µg/mL; and for the S139 ELISA, influenza-A H3N2 (A/Aichi/2/1968) hemagglutinin protein (Sino Biologicals Inc., Cat # 11707-V08H1-100) antigen at a concentration of 2 μg/mL was used. Coated plates were blocked with 1% bovine serum albumin (BSA) (Alfa Aesar, Fraction V, 97%, standard grade, AAj6465530, Haverhill, MA, USA) in PBST for one hour at 37 °C (Bio-World, 1X PBS Buffer Ready-Pack 41620000-3, VWR, 0.05% Tween20, Dublin, OH, USA). 

Both the rabbit and murine plasma samples were diluted in blocking buffer and incubated for 1 h at 37 °C. In-house-purified EBV321, 1219, or S139 was used as the standard. Detection of the captured EBV321 and 1219 was carried out with horseradish peroxidase (HRP)-conjugated anti-rabbit IgG (H + L) goat secondary Ab (Jackson ImmunoResearch, Cat # 111-035-144, West Grove, PA, USA), diluted 1:15,000 in blocking buffer. Detection of the captured S139 was performed with HRP-conjugated goat anti-mouse IgG (H + L) (Jackson ImmunoResearch, Cat # 115-035-062), diluted 1:5000 in 1% BSA-1X PBST. Plates were developed using an ultra-sensitive TMB substrate (Sigma-Aldrich; Cat#T4444, St. Louis, MO, USA), and the reaction was stopped with 2.0 N sulfuric acid (Spectrum Chemical, New Brunswick, NJ, USA). Absorbance was measured at 450 nm using the EPOCH2 BIOTEK microplate plate reader (Agilent Technologies, Santa Clara, CA, USA). Sample calculations were performed based on a linear regression fit using the appropriate standard calibration curve in Microsoft Excel (ver. 2312).

### 2.9. Statistical Analysis

At the start of the experiments, the mice were randomized based on body weight. Serum Ab concentrations were analyzed at multiple timepoints and compared using one-way ANOVA, using the Shapiro–Wilk test for parametric data or the Kruskal–Wallis test for non-parametric data, with Tukey’s/Dunn’s multiple comparisons test. Data from each individual animal are represented as single data points, with the error bars indicating standard deviation from the mean unless otherwise stated. Data with a *p*-value lower than 0.05 were considered to have a statistically significant difference. All image and statistical analyses were performed using GraphPad Prism 10.1.1 (GraphPad Software, San Diego, CA, USA). 

## 3. Results

### 3.1. Hyaluronidase Treatment Improves Serum Antibody Levels following Electroporation of pDNA

Hyaluronidase has previously been used in conjunction with pDNA delivery and electroporation to improve Ab production [26,27]. An experiment was performed to confirm the role of hyaluronidase in enhancing Ab production via gene electrotransfer, in which mice were segregated into two groups. The first experimental group received a pretreatment of 20 µL of hyaluronidase (3U), administered 30 min prior to the pDNA injection and electroporation. In contrast, the control group did not receive any hyaluronidase pretreatment.

**Figure 1:**
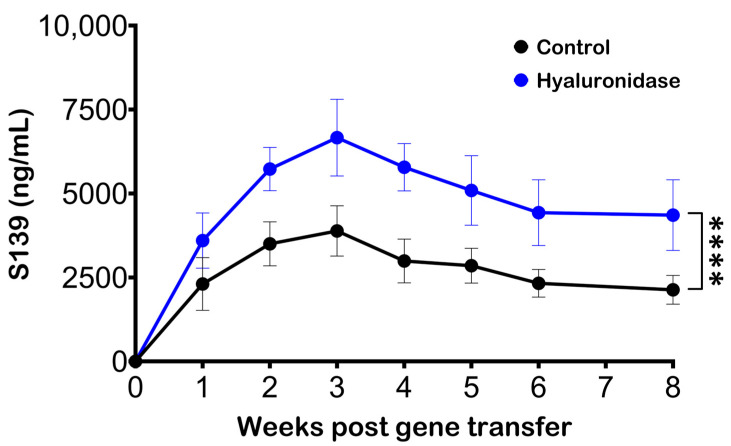
Effect of electroporation site pretreatment with hyaluronidase on serum Ab production in mice. BALB/c mice received intramuscular pDNA electrotransfer of 5 µg pDNA in the TA muscle without (black) or with (blue) 30 min hyaluronidase pretreatment. Blood samples were collected and analyzed by ELISA to quantify Ab levels. Markers indicate the mean expression levels per group (n = 5) and the error bars indicate the standard deviation. The statistical test performed was two-way ANOVA and **** represents *p* < 0.0001.

Serum Ab levels were significantly elevated in the group treated with hyaluronidase, relative to the control group (Figure 1). This increase was not only substantial but also consistently observed at each time point of measurement. The consistently higher Ab production in the hyaluronidase-treated group strongly suggests that the enzymatic pretreatment notably improved the gene electrotransfer process.

### 3.2. pDNA Distribution in Skeletal Muscle

**Figure 2:**
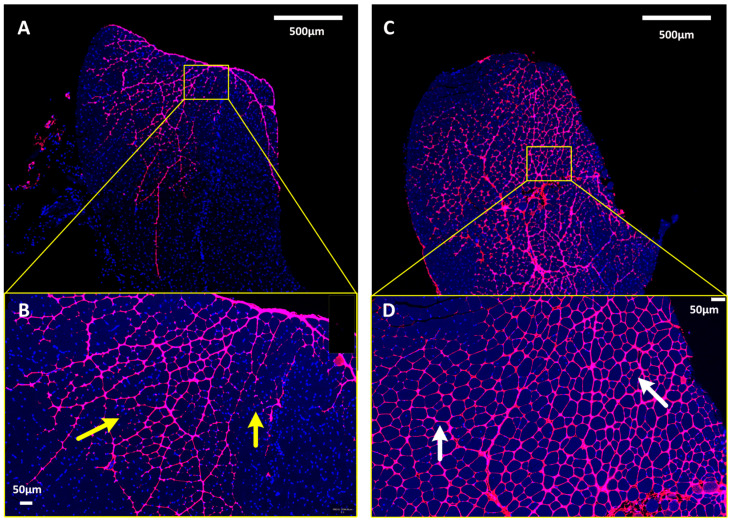
Hyaluronidase treatment affects plasmid distribution in skeletal muscle. (**A**,**B**) The TA muscle in mice was injected with fluorescently labeled pDNA (in magenta) without any hyaluronidase pretreatment. Areas surrounding individual muscle fibers exhibit little to no fluorescence (yellow arrows), suggesting a minimal plasmid presence. (**C**,**D**) The TA muscle in mice was injected with fluorescently labeled pDNA (in magenta) after 5 min of hyaluronidase pretreatment. Areas surrounding individual muscle fibers exhibit uniform fluorescence (white arrows), suggesting a consistent plasmid penetration throughout the muscle fascicles.

Hyaluronidase works by temporarily depolymerizing hyaluronic acid in the ECM, thereby reducing tissue viscosity, and facilitating the dispersion and absorption of injected materials [28]. In a clinical setting, hyaluronidase is used primarily as an adjuvant to increase the dispersion and absorption of injected drugs. We hypothesized that hyaluronidase would enhance Ab production, as exhibited earlier when optimizing the distribution of pDNA within muscle tissue. To test this hypothesis, the TA muscles of mice were administered fluorescently labeled pDNA to explore the differential distribution patterns within muscle tissue, in the absence (Figure 2A,B) or presence (Figure 2C,D) of hyaluronidase pretreatment. 

Notably, pretreatment with hyaluronidase promoted a more homogeneous distribution of the pDNA throughout the targeted muscle tissue. This effect was discernible through the absence of plasmid saturation between fascicle bundles and the observation of consistent plasmid penetration throughout the muscle fascicles. In the absence of hyaluronidase pretreatment, the dispersion pattern of the plasmid was markedly less uniform, appearing uneven and sporadic within the muscle fascicles. Notably, some areas surrounding individual muscle fibers exhibited little to no fluorescence, suggesting minimal plasmid presence. This difference in pDNA distribution patterns reveals the role of hyaluronidase in augmenting the efficacy of plasmid delivery by ensuring a more even plasmid dispersion within the muscle tissue.

### 3.3. Effect of Hyaluronidase Pretreatment on Electroporation Efficiency

The effect of hyaluronidase pretreatment time on the efficacy of plasmid distribution and subsequent transfection efficiency is an important consideration. The incubation time can influence how effectively hyaluronidase modifies the ECM. A short incubation may not allow sufficient time for adequate hyaluronidase activity, while a long incubation might lead to a decrease in its enzymatic activity before the introduction of the target substance (such as pDNA) [29].

An experimental investigation was structured to assess the impact of hyaluronidase pretreatment timing on Ab production following pDNA injection and electroporation in murine and rabbit models. 

**Figure 3:**
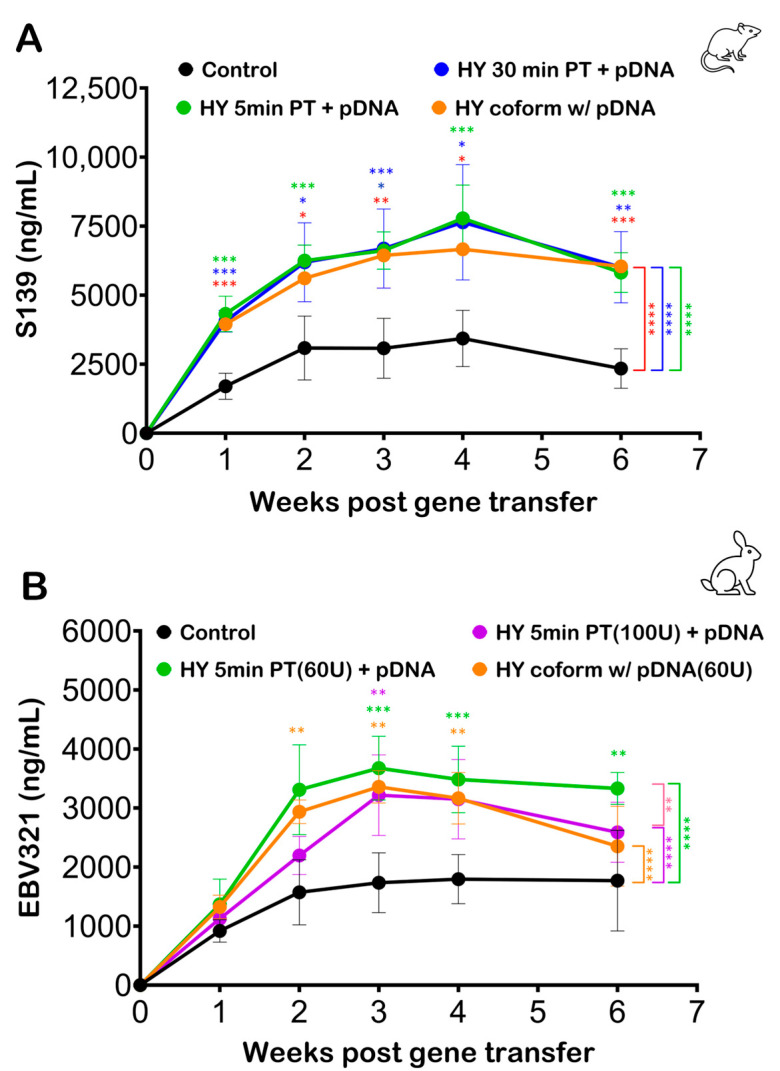
Hyaluronidase pretreatment time does not affect gene electrotransfer in skeletal muscle. (**A**) Mice (*n* = 5 per cohort) were exposed to varying pretreatment times (PTs) of hyaluronidase (HY) before pDNA injection and electroporation. Blood samples were collected and analyzed by ELISA to quantify Ab levels. No significant difference in serum Ab concentration was observed between any of the hyaluronidase treatment groups (**B**) Rabbits (*n* = 5 per group) were exposed to varying pretreatment times as well as doses of hyaluronidase before pDNA injection and electroporation. Blood samples were collected and analyzed by ELISA to quantify serum Ab levels. No significant difference in serum Ab concentration was observed between the 60 U hyaluronidase pretreatment group and the 60 U coformulation group in the rabbit model. Markers indicate average Ab expression levels, and the error bars indicate standard deviation. The statistical test performed was two-way ANOVA, where **** represents *p* < 0.0001, *** represents *p* < 0.001, ** represents *p* < 0.01, and * represents *p* < 0.05.

In the murine model, four groups were evaluated: (1) a 5 min hyaluronidase pretreatment group, (2) a 30 min hyaluronidase pretreatment group, (3) a coformulation group where the pDNA was formulated in hyaluronidase but the plasmid dose, concentration, and amount of hyaluronidase used were maintained consistently with the pretreatment groups, and (4) a control group without any hyaluronidase pretreatment. Despite the different hyaluronidase exposure times in the three treatment groups, there were no statistically significant differences in serum Ab concentrations at any time point (Figure 3A). These results suggest that varying the incubation time of hyaluronidase pretreatment does not significantly affect the serum Ab concentration following electroporation in the murine model.

In the rabbit model study, the amount of hyaluronidase was calculated to be proportional to the amount of pDNA and hyaluronidase delivered in the murine model. The groups included a 5 min hyaluronidase pretreatment, a hyaluronidase coformulation group, a control group without hyaluronidase, and a pretreatment group with a higher concentration of hyaluronidase. Serum Ab concentrations in the rabbit model (Figure 3B) mirrored the murine model findings. No significant difference in serum Ab concentration was observed between the pretreatment and the coformulation groups for the same hyaluronidase concentration (60 U). The increased concentration of hyaluronidase (100 U) also failed to yield any significant improvement in Ab production compared to the standard (60 U) hyaluronidase treatment groups.

In both murine and rabbit models, the data indicate that neither the timing of hyaluronidase pretreatment nor its coformulation with the pDNA significantly influences the overall increase in serum Ab concentration following electroporation. These results suggest that the benefits of hyaluronidase in enhancing pDNA uptake for Ab production are not sensitive to the variations in pretreatment timing or formulation.

### 3.4. Bioimpedance of Skeletal Muscle

Bioimpedance assessments were conducted on murine skeletal muscle to evaluate the changes induced by hyaluronidase treatment. Bioimpedance assessments have two parts: a real impedance characterized as the material’s resistance to the flow of electric current and related to conductance, and an imaginary part defined as the material’s resistance to a change in electric current and related to capacitance in biological materials [30]. Assessment of both the real and imaginary parts of bioimpedance is necessary for comprehensive characterization of the electrical properties of the material. To better understand changes in the muscle impedance, the experimentally obtained curves were fitted to Equation (3) (defined in Section 2.4) using a non-linear least-squares iterative process. Through this process, key parameters such as acpe, A, R_i_, R_e_, C, and alpha from the Cole–Cole model were determined, and subsequently, these parameters were utilized to discern and evaluate variations in muscle morphology under different experimental conditions. This methodological approach offers a deeper insight into the changes in bioelectrical properties of muscle tissue, particularly in the context of gene electrotransfer both prior to and following pDNA injection, and in the presence versus absence of hyaluronidase pretreatment. 

**Figure 4:**
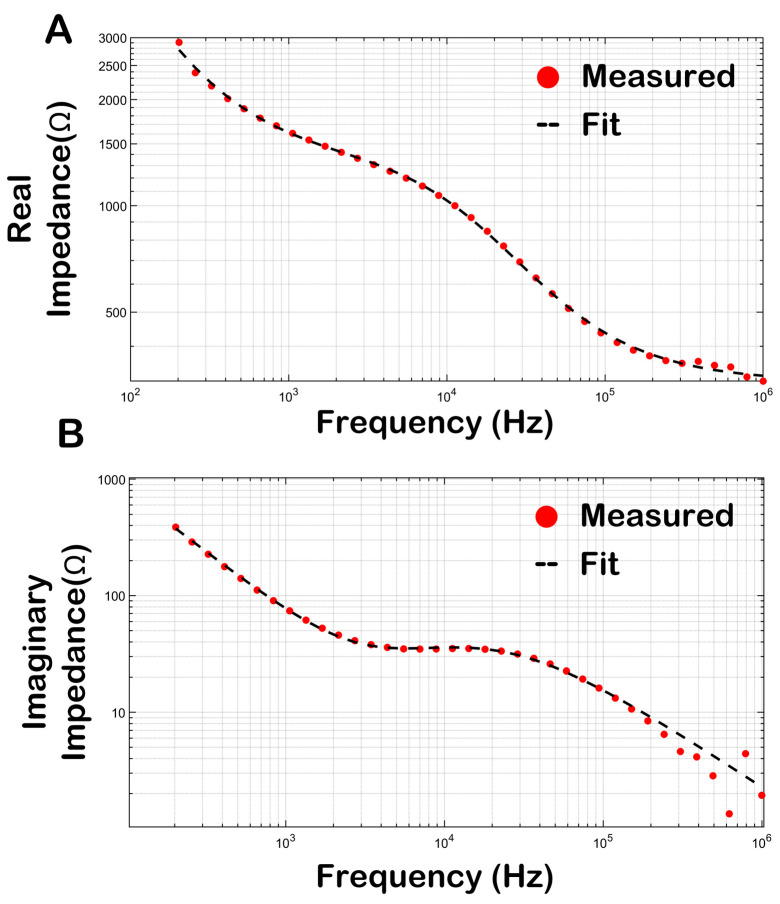
Complex impedance versus frequency (100 Hz–1 MHz) for murine TA muscle. (**A**) Red markers indicate the real part of the experimentally obtained complex impedance, whereas the black dashed line depicts the real part of a simulated curve using parameters obtained by fitting the Cole–Cole model to the experimental curve and plugging them back in to the Cole–Cole model in Equation (3). (**B**) Red markers indicate the imaginary part of the experimentally obtained complex impedance, whereas the black dashed line depicts the imaginary part of a simulated curve using parameters obtained by fitting the Cole–Cole model to the experimental curve and plugging them back in to the Cole–Cole model in Equation (3). Real and imaginary parts of the complex impedance were fitted simultaneously to the Cole–Cole equation using a least-squares iterative process.

**Figure 5:**
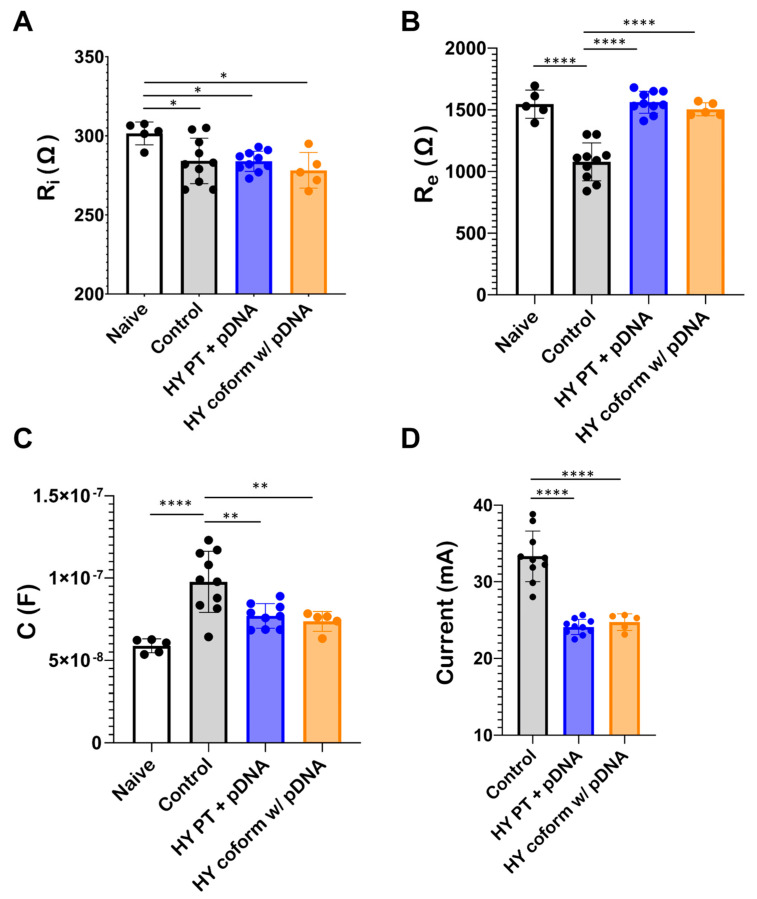
Cole-Cole model parameters (R_i_, R_e_, and C), calculated from fitting experimentally obtained complex impedance data to Cole–Cole equations, vary in response to different experimental conditions. Complex impedance was recorded for the TA muscle of mice without any plasmid injection (naïve), immediately after plasmid injection (control), immediately after plasmid injection into a TA muscle pretreated with hyaluronidase for 5 min (HY 5 min PT + pDNA), and immediately after plasmid coformulated with hyaluronidase was injected (HY coform w/pDNA). (**A**–**C**) Cole–Cole parameters exhibit significant differences following hyaluronidase treatment compared to cases where no hyaluronidase was used, implying changes in muscle morphology upon hyaluronidase treatment. (**D**) Electroporation current recordings from the different experimental conditions exhibit significant differences when the injection site is treated with hyaluronidase, implying that hyaluronidase treatment makes the site less conducive to current flow. Each marker represents data from an individual mouse, with error bars indicating standard deviation of the mean. The statistical test performed was one-way ANOVA, where **** represents *p* < 0.0001, ** represents *p* < 0.01, and * represents *p* < 0.05.

Both the real (Figure 4A) and imaginary (Figure 4B) impedance profiles of murine TA muscle were determined over a frequency range of 100 Hz–1 MHz. The red markers represent the experimentally obtained impedance profile whereas the black dashed lines represent impedance profiles simulated using the parameters obtained from the Cole–Cole model after the iterative fitting process. The Cole–Cole model parameters obtained vary in response to different experimental conditions (Figure 5). Notably, there is a significant change in those parameters between muscle treated with plasmid alone and muscle subjected to plasmid with hyaluronidase treatment. The analysis further revealed no significant differences between muscle pretreated with hyaluronidase and that where hyaluronidase was coformulated with the plasmid in terms of model parameters. 

However, parameters such as R_e_ and C exhibited statistically significant differences when hyaluronidase was incorporated into the treatment. Furthermore, the current during electroporation at a constant voltage decreased for both hyaluronidase treatment groups compared to the control group (Figure 5D). This notable decrease in current in the hyaluronidase-treated samples in addition to the changes in R_e_ and C parameters suggest that hyaluronidase induces changes in muscle morphology, likely through the breakdown of the ECM, leading to increased impedance.

### 3.5. Optimizing Electroporation Parameters following Hyaluronidase Treatment

Building upon prior observations indicating that the use of hyaluronidase results in a statistically significant reduction in the current during electroporation, we further investigated the optimization of electroporation parameters in the presence of hyaluronidase. To this end, pDNA was coformulated with hyaluronidase and the strength of the electric field was varied from 150 V/cm to 300 V/cm in mice. This range was selected to comprehensively assess the impact of escalating electric field strengths on gene electrotransfer efficiency upon hyaluronidase use.

**Figure 6:**
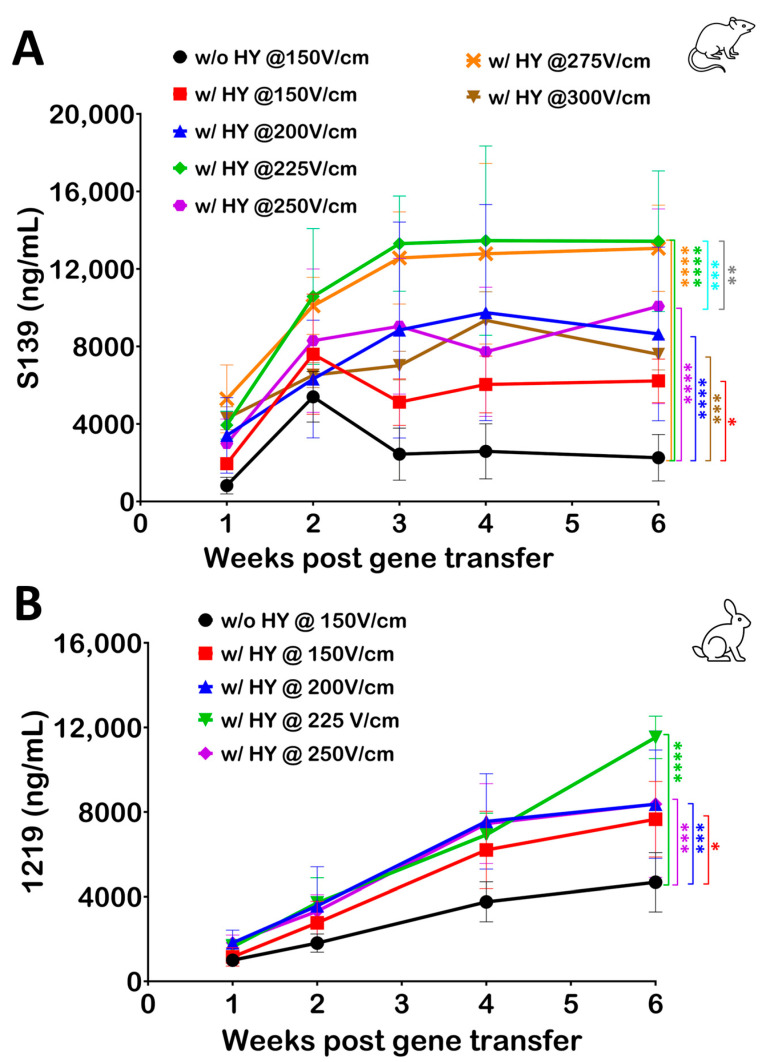
Optimization of electric field strength for electroporation following hyaluronidase treatment. (**A**) Electric field strength for electroporation was varied from 150 V/cm to 300 V/cm in mice following injection of pDNA coformulated with hyaluronidase (HY). Blood samples were collected and analyzed by ELISA to quantify Ab levels. Serum Ab levels indicate an increase in Ab production with escalating electric field strength in mice. (**B**) Similarly, in rabbits, electric field strength for electroporation was varied from 150 V/cm to 250 V/cm following injection of pDNA coformulated with hyaluronidase. Serum Ab levels indicate an increase in Ab production when electric field strength for electroporation is increased from 150 V/cm. Markers indicate average serum Ab levels per group (*n* = 5). Error bars indicate standard deviation. The statistical test performed was two-way ANOVA, where **** represents *p* < 0.0001, *** represents *p* < 0.001, ** represents *p* < 0.01, and * represents *p* < 0.05.

The data revealed a consistent increase in serum Ab levels in response to the escalation of the electric field strength (Figure 6A). Notably, electric field strengths of 225 V/cm and 275 V/cm demonstrated the highest efficiency in gene electrotransfer when used in conjunction with hyaluronidase. A similar study was performed in rabbits where hyaluronidase was coformulated with pDNA (final hyaluronidase dose of 60 U) and the electric field strength was varied from 150 V/cm to 250 V/cm. Serum Ab levels revealed a similar effect in rabbits as was observed in mice, where increasing the electric field strength beyond 150 V/cm yielded an increase in Ab (Figure 6B).

Serum Ab levels in both animal models revealed that a drop in current due to hyaluronidase use can be compensated for with an increase in electric field strength to further enhance the efficiency. This finding not only informs the optimization of electroporation protocols but also underscores the importance of tailored electric field parameters in enhancing the effectiveness of hyaluronidase in gene electrotransfer applications.

## 4. Discussion

The use of gene transfer techniques in skeletal muscle has emerged as a promising strategy for various applications, including gene replacement, the production of therapeutic proteins for systemic release, and genetic vaccination [31]. Skeletal muscle has been utilized for DNA-based therapeutic protein production using different vectors such as adenoviruses and retroviruses, as well as the direct introduction of pDNA without a carrier [32]. Viral vectors face limitations due to the development of antibodies specific to the vector, which can hinder repeated administrations and, in some cases, trigger harmful immune responses [33,34]. In contrast, naked pDNA offers certain advantages, including the ease of creating new genetic constructs and the ability to administer them repeatedly in animals with functional immune systems, as well as in humans. However, a notable challenge associated with pDNA delivery into muscle tissue is its relatively low transfection efficiency following injection. Recently, this issue has been addressed through the development of in vivo electrotransfer techniques, which have consistently led to a remarkable 10- to 100-fold increase in gene expression levels [35,36].

One of the main challenges in delivering pDNA into target cells is overcoming the extracellular barriers. The ECM and interstitial fluid can impede the movement of pDNA and, therefore, its ability to reach a sufficient number of target cells. Indeed, a major hurdle to achieving increased gene expression levels is the poor plasmid distribution upon injection because the ECM hinders the uniform distribution of the plasmid around the muscle fibers [37]. Hyaluronidase is an enzyme that has been hypothesized to help improve the distribution of pDNA by breaking down hyaluronic acid, a major component of the ECM, thereby making it less viscous and facilitating improved diffusion of the pDNA [38]. Hyaluronidase’s ability to break down the ECM can be especially important when delivering genetic material to tissues with a dense ECM, such as muscle tissue. By facilitating the distribution of pDNA, hyaluronidase can ultimately lead to an increased transfection efficiency. This means a higher percentage of target cells can take up and express the introduced genetic material, improving the overall effectiveness of the treatment.

It is noteworthy that hyaluronidase has previously been demonstrated to have utility in enhancing the efficiency of in vivo gene transfer methods, including both virus-based and gene-electrotransfer-based techniques [39,40]. In agreement with these data, our results revealed a notable enhancement in gene electrotransfer efficiency when hyaluronidase treatment was applied. Specifically, animals subjected to hyaluronidase treatment exhibited a remarkable two-fold increase in serum protein expression levels compared to those without hyaluronidase pretreatment. Our investigation also focused on assessing the impact of hyaluronidase on the distribution of pDNA within skeletal muscle tissue. Indeed, the data reveal that pretreatment with hyaluronidase leads to a more uniform and homogeneous distribution of pDNA throughout the targeted muscle tissue, and this translates to improved electroporation efficiency and higher antibody expression. These outcomes emphasize the valuable role of hyaluronidase in augmenting the efficiency of gene transfer procedures and highlight its potential for enhancing therapeutic applications in gene therapy and related fields.

Conventionally, hyaluronidase is pre-administered 1–4 h prior to pDNA delivery in both murine [26,27,41,42,43] and rat [44,45] models. This pretreatment is employed to allow sufficient time for the enzyme to exert its effects on the ECM, which is essential for achieving the desired outcome. However, this approach necessitates anesthetizing the animals twice to ensure proper localization of the enzyme in the target muscle, which can induce stress and is logistically cumbersome for the operator. Recent findings have challenged the conventional timing of hyaluronidase pretreatment. Studies have indicated that a significant and comparable impact can be achieved when the muscle is pretreated with hyaluronidase just 10 min before plasmid injection [26]. This suggests that the beneficial effects of hyaluronidase can be rapidly realized. Supporting this notion, research by Akerstrom et al. [19] demonstrated that even pre-injecting hyaluronidase merely 1 min prior to plasmid delivery is as effective as the conventional 10 min pretreatment. 

In our investigations encompassing both murine and rabbit models, we explored the timing of muscle pretreatment with hyaluronidase. While pDNA coformulated with hyaluronidase has been previously used in gene electrotransfer applications [46,47,48], its efficacy compared to hyaluronidase pretreatment has not been investigated. Notably, we observed no significant differences in protein production levels when pretreating the muscle with hyaluronidase either 30 min or 5 min prior to plasmid injection and electroporation. Furthermore, we investigated the effect of coformulating hyaluronidase with the plasmid, thereby delivering them simultaneously into the target muscle. This approach yielded comparable protein expression levels to hyaluronidase pretreatment (see Figure 3). Using a coformulated plasmid complex not only reduces stress for the animals and streamlines the procedure for operators but also ensures the colocalization of hyaluronidase and the plasmid within the target tissue, potentially optimizing the desired therapeutic effects and allowing for a more controlled and precise delivery of the pDNA intramuscularly. Additionally, a coformulated plasmid complex minimizes the burden of repeated injections for individuals undergoing such treatments. 

Bioimpedance measurements have become an indispensable tool in the comprehensive assessment of biological materials and tissues [49,50,51,52], including skeletal muscle physiology, providing valuable insights into muscle composition, hydration status, and contractile properties [53,54,55]. By analyzing electrical impedance within skeletal muscle tissue, bioimpedance techniques offer a non-invasive means of exploring various facets of muscle health and function. These measurements find applications in diverse fields, from sports science and clinical diagnostics to rehabilitation [56,57,58]. In this study, we used bioimpedance measurements to detect and quantify changes in muscle morphology induced by the administration of hyaluronidase. We used a Cole–Cole impedance model to establish a mathematical representation of the bioimpedance recorded from muscle tissues. The Cole–Cole model provides a robust and accurate framework for assessing complex impedances and is specifically tailored to biological tissues, including skeletal muscle, for which it can capture the multifrequency impedance response accurately. Unlike simpler models, such as the resistor–capacitor (RC) model [50], the Cole–Cole model considers the dispersion of impedance across a range of frequencies and is particularly relevant for biological tissues, which exhibit frequency-dependent impedance behavior due to the presence of cellular and structural elements with varying electrical properties. Parameters such as alpha (α) can reveal the heterogeneity of muscle fibers, while R_i_ (R-intracellular) and R_e_ (R-extracellular) provide information about intracellular and extracellular properties, respectively. Additionally, C in conjunction with R_i_ and R_e_ relate to a tissue’s relaxation dynamics, and the transition between extracellular and intracellular current pathways.

In our studies, we used bioimpedance measurements to gain an insight into changes in the skeletal muscle upon hyaluronidase injection. Cole–Cole model parameters, especially R_e_ and C, exhibit a clear distinction between conditions without plasmid injection, samples with plasmid injection, and samples with coformulated pDNA, indicating changes in the intracellular and extracellular properties of muscle tissue upon hyaluronidase injection. Hyaluronidase reduced tissue conductivity, as exhibited by changes in these parameters. As hyaluronidase breaks down HA, it disrupts the structural integrity of the ECM. The ECM’s ability to maintain its gel-like consistency and hydration is compromised, leading to a reduction in the ECM’s viscosity and its ability to trap water and ions, including electrolytes [59]. Since electrical conductivity in tissues relies on the presence of ions in the extracellular fluid, their decrease leads to a reduction in tissue electrical conductivity, as seen in our bioimpedance and electrical recordings.

The vast majority of studies conducted over the last three decades have focused on optimizing parameters related to the use of hyaluronidase, such as the hyaluronidase dose and the time interval between the injections of hyaluronidase and pDNA [19,20,26]. In addition to bioimpedance parameters, changes in the current levels during constant-voltage electroporation also indicate the change in tissue conductivity when hyaluronidase is introduced. This information is valuable as it guides us towards optimizing electroporation treatment strategies to ensure safety and tolerability as well as increase the efficiency of an electroporation procedure. To compensate for the reduced muscle conductivity while performing electroporation, an increase in electric field strength was evaluated in both the murine and rabbit animal models. As evident in the mice model, a further increase in the field strength results in a drop in Ab production. A possible explanation for this reduction in Ab expression is the disruption of cellular processes and structures due to the excessive stress imposed by the electric field. High electric field strengths can induce cellular damage via a wide variety of mechanisms such as membrane damage, ATP depletion, or mitochondrial damage, leading to impaired protein synthesis machinery and production [12,13]. Improved levels of protein production emphasize the importance of optimizing electroporation parameters upon hyaluronidase treatment to further improve its benefits. 

The incorporation of hyaluronidase into pDNA electroporation offers several distinct advantages, significantly improving the overall efficiency of the procedure in multiple ways. These enhancements contribute to more efficient protein production, ultimately facilitating the achievement of therapeutic goals. When hyaluronidase is employed alongside pDNA electroporation, one of the most notable benefits is the substantial improvement in total protein production. Hyaluronidase acts as a facilitator by breaking down hyaluronic acid in the ECM, thus reducing tissue resistance to the passage of molecules. This breakdown enhances the distribution and uptake of pDNA, allowing a greater number of cells to successfully incorporate the genetic material and produce the therapeutic protein of interest. The use of hyaluronidase and understanding the mechanism of its impact on protein production can offer new perspectives on improving the distribution of pDNA in skeletal muscle. Techniques can be developed to mimic hyaluronidase’s mechanism of action, thereby enhancing the pDNA distribution and transfection efficiency without the need for an enzyme. This approach could potentially improve the biodistribution and transfection efficiency without enzymatic intervention or the limitations associated with enzyme formulations. Additionally, the inclusion of hyaluronidase in the electroporation process offers a strategic advantage in terms of optimizing dosages. By incorporating hyaluronidase, therapeutic levels of proteins can potentially be achieved while using lower doses of pDNA. This reduction in the required DNA dosage will not only conserve valuable material but also minimize potential side effects or complications associated with high pDNA doses. Finally, another compelling advantage of hyaluronidase-assisted pDNA electroporation is its ability to facilitate therapeutic protein production even at lower electric field strengths. In the absence of hyaluronidase, attaining therapeutic levels of protein production may require the application of higher electric field strengths, as an increasing electric field strength is correlated with an increasing amount of protein production [60,61]. However, such an electric field strength could bring about potential risks or discomfort during the delivery process. The significance of hyaluronidase becomes apparent as it plays a crucial role in improving the efficiency of DNA delivery. By facilitating enhanced DNA delivery efficiency, hyaluronidase can potentially enable the achievement of effective therapeutic protein expression even at lower electric field strengths. This not only enhances patient safety and comfort but also extends the range of applicability for electroporation-based therapies, as it broadens the spectrum of tissues and conditions where this technique can be effectively employed.

The utilization of hyaluronidase in pDNA electroporation has the potential to yield multiple benefits in regard to the application of this important technique. By enhancing the total protein production, reducing the required DNA dosage, and enabling therapeutic protein expression at lower electric field strengths, hyaluronidase can play a pivotal role in optimizing the pDNA-based delivery of therapeutic proteins. These benefits have the potential to make it a valuable tool in various biomedical and clinical applications, offering greater efficiency and control over the DNA dosage as well as the requisite electric field strength for achieving the therapeutic outcomes. Moreover, comprehending hyaluronidase’s mechanism of action in improving the pDNA distribution can pave the way for a deeper understanding of the efficient distribution of pDNA within skeletal muscle, ultimately facilitating the development of other non-enzymatic ways of achieving an optimized distribution of pDNA and its increased uptake by cells.

## Data Availability

The data can be shared upon request.

## References

[1] Wolff J.A., Malone R.W., Williams P., Chong W., Acsadi G., Jani A., Felgner P.L. (1990). Direct Gene Transfer into Mouse Muscle in Vivo. Science.

[2] Sung Y., Kim S. (2019). Recent advances in the development of gene delivery systems. Biomater. Res..

[3] Al-Dosari M.S., Gao X. (2009). Nonviral Gene Delivery: Principle, Limitations, and Recent Progress. AAPS J..

[4] Felgner P.L., Gadek T.R., Holm M., Roman R., Chan H.W., Wenz M., Northrop J.P., Ringold G.M., Danielsen M. (1987). Lipofection: A highly efficient, lipid-mediated DNA-transfection procedure. Proc. Natl. Acad. Sci. USA.

[5] Boussif O., Lezoualc’h F., Zanta M.A., Mergny M.D., Scherman D., Demeneix B., Behr J.P. (1995). A versatile vector for gene and oligonucleotide transfer into cells in culture and in vivo: Polyethylenimine. Proc. Natl. Acad. Sci. USA.

[6] Nathwani A.C., Tuddenham E.G.D., Rangarajan S., Rosales C., McIntosh J., Linch D.C., Chowdary P., Riddell A., Pie A.J., Harrington C. (2011). Adenovirus-Associated Virus Vector–Mediated Gene Transfer in Hemophilia B. N. Engl. J. Med..

[7] Dull T., Zufferey R., Kelly M., Mandel R.J., Nguyen M., Trono D., Naldini L. (1998). A Third-Generation Lentivirus Vector with a Conditional Packaging System. J. Virol..

[8] Neumann E., Schaefer-Ridder M., Wang Y., Hofschneider P.H. (1982). Gene transfer into mouse lyoma cells by electroporation in high electric fields. EMBO J..

[9] Aihara H., Miyazaki J. (1998). Gene transfer into muscle by electroporation in vivo. Nat. Biotechnol..

[10] Meaking W.S., Edgerton J., Wharton C.W., Meldrum R.A. (1995). Electroporation-induced damage in mammalian cell DNA. Biochim. Biophys. Acta (BBA)-Gene Struct. Expr..

[11] Weaver J.C. (1993). Electroporation: A general phenomenon for manipulating cells and tissues. J. Cell. Biochem..

[12] Napotnik T.B., Polajžer T., Miklavčič D. (2021). Cell death due to electroporation—A review. Bioelectrochemistry.

[13] Gehl J. (2003). Electroporation: Theory and methods, perspectives for drug delivery, gene therapy and research. Acta Physiol. Scand..

[14] Kandušer M., Ušaj M. (2014). Cell electrofusion: Past and future perspectives for antibody production and cancer cell vaccines. Expert Opin. Drug Deliv..

[15] Weaver J.C., Chizmadzhev Y.A. (1996). Theory of electroporation: A review. Bioelectrochem. Bioenerg..

[16] De Robertis M., Pasquet L., Loiacono L., Bellard E., Messina L., Vaccaro S., Di Pasquale R., Fazio V.M., Rols M.P., Teissie J. (2018). In Vivo Evaluation of a New Recombinant Hyaluronidase to Improve Gene Electro-Transfer Protocols for DNA-Based Drug Delivery against Cancer. Cancers.

[17] Andrews C.D., Luo Y., Sun M., Yu J., Goff A.J., Glass P.J., Padte N.N., Huang Y., Ho D.D. (2017). In Vivo Production of Monoclonal Antibodies by Gene Transfer via Electroporation Protects against Lethal Influenza and Ebola Infections. Mol. Ther. Methods Clin. Dev..

[18] Yu Y., Lee P., Ke Y., Zhang Y., Yu Q., Lee J., Li M., Song J., Chen J., Dai J. (2010). A Humanized Anti-VEGF Rabbit Monoclonal Antibody Inhibits Angiogenesis and Blocks Tumor Growth in Xenograft Models. PLoS ONE.

[19] Akerstrom T., Vedel K., Needham J., Hojman P., Kontou E., Hellsten Y., Wojtaszewski J.F. (2015). Optimizing hyaluronidase dose and plasmid DNA delivery greatly improves gene electrotransfer efficiency in rat skeletal muscle. Biochem. Biophys. Rep..

[20] Peri D., Deville M., Poignard C., Signori E., Natalini R. (2020). Numerical optimization of plasmid DNA delivery combined with hyaluronidase injection for electroporation protocol. Comput. Methods Programs Biomed..

[21] Cole Kenneth S. (1940). Permeability and Impermeability of Cell Membranes for Ions. Cold Spring Harb. Symp. Quant. Biol..

[22] Freeborn Todd J. (2013). A Survey of Fractional-Order Circuit Models for Biology and Biomedicine. IEEE J. Emerg. Sel. Top. Circuits Syst..

[23] Egholm M., Christensen L., Deuholm K.L., Buchardt O., Coull J., Nielsen P.E. (1995). Efficient pH-independent sequence-specific DNA binding by pseudoisocytosine-containing bis-PNA. Nucleic Acids Res..

[24] Zelphati O., Liang X., Hobart P., Felgner P.L. (1999). Gene Chemistry: Functionally and Conformationally Intact Fluorescent Plasmid DNA. Hum. Gene Ther..

[25] Zelphati O., Liang X., Nguyen C., Barlow S., Sheng S., Shao Z., Felgner P. (2000). PNA-Dependent Gene Chemistry: Stable Coupling of Peptides and Oligonucleotides to Plasmid DNA. BioTechniques.

[26] Mennuni C., Calvaruso F., Zampaglione I., Rizzuto G., Rinaudo D., Dammassa E., Ciliberto G., Fattori E., La Monica N. (2002). Hyaluronidase Increases Electrogene Transfer Efficiency in Skeletal Muscle. Hum. Gene Ther..

[27] McMahon J.M., Signori E., Wells K.E., Fazio V.M., Wells D.J. (2001). Optimisation of electrotransfer of plasmid into skeletal muscle by pretreatment with hyaluronidase–increased expression with reduced muscle damage. Gene Ther..

[28] Buhren B.A., Schrumpf H., Hoff N.-P., Bölke E., Hilton S., Gerber P.A. (2016). Hyaluronidase: From clinical applications to molecular and cellular mechanisms. Eur. J. Med. Res..

[29] DeLorenzi C. (2017). New High Dose Pulsed Hyaluronidase Protocol for Hyaluronic Acid Filler Vascular Adverse Events. Aesthet. Surg. J..

[30] Naranjo-Hernández D., Reina-Tosina J., Min M. (2019). Fundamentals, Recent Advances, and Future Challenges in Bioimpedance Devices for Healthcare Applications. J. Sens..

[31] Pagant S., Liberatore R.A. (2021). In Vivo Electroporation of Plasmid DNA: A Promising Strategy for Rapid, Inexpensive, and Flexible Delivery of Anti-Viral Monoclonal Antibodies. Pharmaceutics.

[32] Quigley A., Lowes K., Kornberg A.J., Cook M.J., Kapsa R. (2006). Therapeutic DNA Delivery to Skeletal Muscle. Curr. Genom..

[33] Bessis N., GarciaCozar F.J., Boissier M.-C. (2004). Immune responses to gene therapy vectors: Influence on vector function and effector mechanisms. Gene Ther..

[34] Thomas C.E., Ehrhardt A., Kay M.A. (2003). Progress and problems with the use of viral vectors for gene therapy. Nat. Rev. Genet..

[35] Mir L.M., Bureau M.F., Gehl J., Rangara R., Rouy D., Caillaud J.M., Delaere P., Branellec D., Schwartz B., Scherman D. (1999). High-efficiency gene transfer into skeletal muscle mediated by electric pulses. Proc. Natl. Acad. Sci. USA.

[36] Mathiesen I. (1999). Electropermeabilization of skeletal muscle enhances gene transfer in vivo. Gene Ther..

[37] Bureau M.F., Naimi S., Ibad R.T., Seguin J., Georger C., Arnould E., Maton L., Blanche F., Delaere P., Scherman D. (2004). Intramuscular plasmid DNA electrotransfer: Biodistribution and degradation. Biochim. Biophys. Acta (BBA)-Gene Struct. Expr..

[38] Wohlrab J., Wohlrab D., Wohlrab L., Wohlrab C., Wohlrab A. (2014). Use of Hyaluronidase for Pharmacokinetic Increase in Bioavailability of Intracutaneously Applied Substances. Ski. Pharmacol. Physiol..

[39] Gollins H., McMahon J., Wells K.E., Wells D.J. (2003). High-efficiency plasmid gene transfer into dystrophic muscle. Gene Ther..

[40] Favre D., Cherel Y., Provost N., Blouin V., Ferry N., Moullier P., Salvetti A. (2000). Hyaluronidase enhances recombinant adeno-associated virus (rAAV)-mediated gene transfer in the rat skeletal muscle. Gene Ther..

[41] Chiarella P., Santis S.D., Fazio V.M., Signori E. (2013). Hyaluronidase contributes to early inflammatory events induced by electrotransfer in mouse skeletal muscle. Hum. Gene Ther..

[42] Molnar M.J., Gilbert R., Lu Y., Liu A.-B., Guo A., Larochelle N., Orlopp K., Lochmuller H., Petrof B.J., Nalbantoglu J. (2004). Factors Influencing the Efficacy, Longevity, and Safety of Electroporation-Assisted Plasmid-Based Gene Transfer into Mouse Muscles. Mol. Ther..

[43] Schertzer J.D., Plant D.R., Lynch G.S. (2006). Optimizing Plasmid-Based Gene Transfer for Investigating Skeletal Muscle Structure and Function. Mol. Ther..

[44] Bruce C.R., Brolin C., Turner N., Cleasby M.E., van der Leij F.R., Cooney G.J., Kraegen E.W. (2007). Overexpression of carnitine palmitoyltransferase I in skeletal muscle in vivo increases fatty acid oxidation and reduces triacylglycerol esterification. Am. J. Physiol.-Endocrinol. Metab..

[45] Bruce C.R., Hoy A.J., Turner N., Watt M.J., Allen T.L., Carpenter K., Cooney G.J., Febbraio M.A., Kraegen E.W. (2009). Overexpression of carnitine palmitoyltransferase-1 in skeletal muscle is sufficient to enhance fatty acid oxidation and improve high-fat diet-induced insulin resistance. Diabetes.

[46] Hollevoet K., Thomas D., Compernolle G., Vermeire G., De Smidt E., De Vleeschauwer S., Smith T.R.F., Fisher P.D., Dewilde M., Geukens N. (2022). Clinically relevant dosing and pharmacokinetics of DNA-encoded antibody therapeutics in a sheep model. Front. Oncol..

[47] Schommer N.N., Nguyen J., Yung B.S., Schultheis K., Muthumani K., Weiner D.B., Humeau L., Broderick K.E., Smith T.R. (2019). Active Immunoprophylaxis and Vaccine Augmentations Mediated by a Novel Plasmid DNA Formulation. Hum. Gene Ther..

[48] Wise M.C., Xu Z., Tello-Ruiz E., Beck C., Trautz A., Patel A., Elliott S.T., Chokkalingam N., Kim S., Kerkau M.G. (2020). In vivo delivery of synthetic DNA–encoded antibodies induces broad HIV-1–neutralizing activity. J. Clin. Investig..

[49] Foster K.R., Schwan H.P. (1989). Dielectric properties of tissues and biological materials: A critical review. Crit. Rev. Biomed. Eng..

[50] Gabriel C., Gabriel S., Corthout E. (1996). The dielectric properties of biological tissues: I. Literature survey. Phys. Med. Biol..

[51] Suster M.A., Vitale N.H., Maji D., Mohseni P. (2016). A Circuit Model of Human Whole Blood in a Microfluidic Dielectric Sensor. IEEE Trans. Circuits Syst. II Express Br..

[52] Maji D., De La Fuente M., Kucukal E., Sekhon U.D.S., Schmaier A.H., Gupta A.S., Gurkan U.A., Nieman M.T., Stavrou E.X., Mohseni P. (2018). Assessment of whole blood coagulation with a microfluidic dielectric sensor. J. Thromb. Haemost..

[53] Castizo-Olier J., Irurtia A., Jemni M., Carrasco-Marginet M., Fernández-García R., Rodríguez F.A. (2018). Bioelectrical impedance vector analysis (BIVA) in sport and exercise: Systematic review and future perspectives. PLoS ONE.

[54] Kyle U.G., Bosaeus I., De Lorenzo A.D., Deurenberg P., Elia M., Gomez J.M., Heitmann B.L., Kent-Smith L., Melchior J.-C., Pirlich M. (2004). Bioelectrical impedance analysis—Part I: Review of principles and methods. Clin. Nutr..

[55] Brown L., Weir J., Brown L., Weir D. (2001). ASEP Procedures Recommendation I: Accurate Assessment of Muscular Strength and Power. J. Exerc. Physiol. Online.

[56] Fu B., Freeborn T.J. (2020). Cole-impedance parameters representing biceps tissue bioimpedance in healthy adults and their alterations following eccentric exercise. J. Adv. Res..

[57] Sato H., Nakamura T., Kusuhara T., Kenichi K., Kuniyasu K., Kawashima T., Hanayama K. (2020). Effectiveness of impedance parameters for muscle quality evaluation in healthy men. J. Physiol. Sci. JPS.

[58] Rutkove S.B., Callegari S., Concepcion H., Mourey T., Widrick J., Nagy J.A., Nath A.K. (2023). Electrical impedance myography detects age-related skeletal muscle atrophy in adult zebrafish. Sci. Rep..

[59] Eikenes L., Tari M., Tufto I., Bruland Ø.S., Davies C.D.L. (2005). Hyaluronidase induces a transcapillary pressure gradient and improves the distribution and uptake of liposomal doxorubicin (Caelyx^TM^) in human osteosarcoma xenografts. Br. J. Cancer.

[60] Hartikka J., Sukhu L., Buchner C., Hazard D., Bozoukova V., Margalith M., Nishioka W.K., Wheeler C.J., Manthorp M., Sawdey M. (2001). Electroporation-Facilitated Delivery of Plasmid DNA in Skeletal Muscle: Plasmid Dependence of Muscle Damage and Effect of Poloxamer 188. Mol. Ther..

[61] Li F., Yamaguchi K., Okada K., Matsushita K., Enatsu N., Chiba K., Yue H., Fujisawa M. (2013). Efficient Transfection of DNA into Primarily Cultured Rat Sertoli Cells by Electroporation1. Biol. Reprod..

